# Toward the cognitive modeling of dynamic decision making

**DOI:** 10.3758/s13423-025-02814-2

**Published:** 2026-04-02

**Authors:** Will Deng, David Kellen, Jared M. Hotaling

**Affiliations:** 1 Department of Psychology, University of Illinois at Urbana-Champaign, Champaign, IL 61820 USA; 2 Department of Psychology, Syracuse University, Syracuse, NY USA

**Keywords:** Dynamic decision making, Decision field theory, Cognitive models, Backward induction

## Abstract

Although most research into risky decision making has focused on simple scenarios – where isolated choices are made independent of one another – many important decisions in life play out across sequences of interdependent events and actions. Despite the ubiquity and importance of such decision problems, we know relatively little about how people manage the complexities of dynamic, multistage decisions. Our work combines techniques from two research traditions to investigate how people handle the challenges of dynamic decision making. We use true-and-error models to estimate the distribution and stability of preference profiles, and the presence of errors. In a complementary analysis we use cognitive modeling based on the Decision Field Theory to investigate the psychological processes underlying dynamic decision making. Decision Field Theory provides a unified framework for testing competing hypotheses about how people collect information and plan for the future. Results from both sets of analyses identify distinct groups of individuals. We discuss the behavioral and cognitive factors distinguishing groups from one another, including degree of planning, strategy shifts, biased information sampling, and effort-saving information processing.

**Supplementary Information:**

The online version contains supplementary material available at 10.3758/s13423-025-02814-2.

## Introduction

Many important life decisions involve sequences of interdependent events and actions. For example, a student planning their career must consider which university to attend, what classes to take, and which internship or job opportunities these might lead to. Each step in the sequence involves risk and uncertainty, influenced by both future actions and external factors. Throughout this process, decision makers must consider events both within their control (e.g., their own potential future choices) and those outside of it (e.g., uncertain events).

That said, most research on risky decision making has focused on simpler “static” scenarios, neglecting the dynamic complexities of multistage decisions. This limited attention has led to disjoint investigations with disparate orientations, goals, and modeling approaches (Bone et al., 2009; Hey & Knoll, 2007; Hey & Lotito, 2009; Hotaling et al., 2015; Hotaling & Kellen, 2022). The present work is an attempt to connect some of these different strands of research on dynamic decision making, leveraging data from a new experiment to develop and test different accounts of the cognitive processes presumed to underlie human choices.

**Fig. 1:**
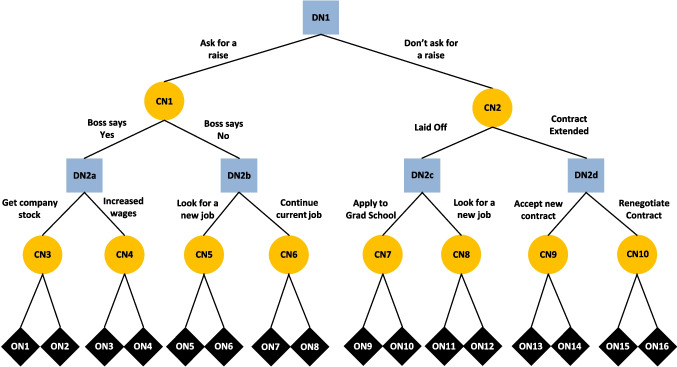
A multistage decision scenario, represented as a decision tree. Decision nodes (DNs) represent points where a decision maker chooses an action. Chance nodes (CNs) represent points where an uncertain external event occurs. Outcome nodes (ONs) represent final outcomes or payoffs

Figure 1 illustrates the kind of multistage decision problem – a decision tree – typically found in this line of research. Decision trees are comprised of three types of nodes: Decision nodes (DNs) are points where a decision maker chooses from the available actions; chance nodes (CNs) are points where some risky or uncertain event (outside of the decision maker’s control) determines the direction of movement; and lastly, the outcome nodes (ONs) represent final consequences.

The decision tree illustrated in Fig. 1 represents a hypothetical scenario faced by an office worker, Emma. Beginning at DN1, Emma must choose whether to ask her boss for a pay raise. If she does – which corresponds to moving leftward to CN1 – she faces uncertainty. Specifically, if her boss says yes, she will face an additional choice between receiving company stock (CN3) or increased wages (CN4). If her boss says no, she must then choose to either look for a new job (CN5) or continue with her current job (CN6). Each of Emma’s final choice alternatives (CN3-10) are represented as CNs because she does not know for sure what outcomes will ultimately result from them.

To understand people’s behavior in dynamic, multi-stage scenarios such as the one illustrated in Fig. 1, behavioral researchers often appeal to normative benchmarks, using the observed deviations from them to motivate and inform the development of new theorical accounts (see Kellen, 2019; Sugden, 2005). In the case of dynamic decision making, *backward induction* is widely accepted as the *optimal strategy* (Bertsekas, 1996; DeGroot, 1970; Von Winterfeldt & Edwards, 1986). This strategy consists of working backwards from the end of a decision tree, planning future actions in reverse temporal order. When planning a future decision, one selects the alternative with the highest expected utility; this value is then associated with that DN and carried up the decision tree when evaluating the higher DNs. The unchosen alternatives are effectively pruned from the tree because the decision maker’s plan renders them irrelevant. The process repeats for all DNs until the beginning of the decision tree is reached. Following the prescriptions of backward induction maximizes one’s overall expected utility.

Backward induction requires that two consistency axioms are satisfied. The first one, *dynamic consistency*, stipulates that decision makers follow through on planned decisions for DN2^1^ because these plans determine which branches are pruned and which values are propagated back through the tree. The second,* consequential consistency,* dictates that decisions should be based solely on future consequences, such that plans about the future decisions made at a given point do not consider the events that led to that point (i.e., information about preceding nodes). Violation of either consistency axiom therefore undermines the viability of backward induction as a descriptor of human decision making.

The descriptive adequacy of backward induction has been challenged by a number of behavioral studies. Busemeyer et al. (2000) found that participants tended to plan riskier choices than the ones that they actually implemented, violating dynamic consistency. Subsequent work by Johnson and Busemeyer (2001) found that the rates with which these violations occurred increased with the length of decision trees (cf. Barkan & Busemeyer, 1999, 2003).

### Modeling dynamic decision making

The model-based characterization of dynamic decision making can be divided into two main modeling approaches. The first involves the development and testing of models that explicitly capture the cognitive processes involved in these decisions. Specifically, participants are placed in decision environments in which they can be shown to violate consistency axioms. The observed violations of axioms provide the “signal” upon which different process-level hypotheses can be formulated and ultimately tested (e.g., Busemeyer et al., 2011). One such process model is *Decision Field Theory*-*Planning* (DFT; Hotaling, 2020; see also Busemeyer & Townsend, 1993) that, due to its vulnerability to different types of biases, can lead to numerous axiom violations – dynamic consistency included. This model is discussed in detail below. Researchers unfamiliar with dynamic decision making will find an analogous modeling approach in the domain of *static* decision making, where violations of Expected Utility Theory – so-called choice paradoxes – have long been used to develop new theoretical accounts such as Prospect Theory (Kahneman & Tversky, 1979), Regret Theory (Loomes & Sugden, 1982), and even Decision Field Theory (Bhatia, 2014; Kellen et al., 2020).

A separate modeling approach, often referred to as measurement modeling, eschews detailed process-level commitments and instead attempts to provide a more agnostic characterization that speaks to the assumptions made by large families of theories (e.g., Birnbaum, 2008; Karabatsos, 2005; McCausland & Marley, 2014). A class of models that exemplifies this approach is the class of *True-and-error models* (TE models; Birnbaum, 2013). Granting the possibility of erroneous responses, TE models characterize the proportion of individuals that conform to different preference patterns. The processes that could underlie these preference profiles are purposely left undefined. This is an important feature of TE models because we do not want to commit to a specific strategy being undertaken, but rather leave that open for more fine-grained accounts to address (e.g., the mental simulation proposed by DFT). TE models can be used to test hypotheses about the presence of specific preference profiles that are theoretically relevant (e.g., are there people in the sample who truly hold intransitive preferences? See Birnbaum & Schmidt, 2008). These hypotheses are directly tested by fixing the proportion of individuals holding those preference profiles to zero and checking whether any aspects of the data originally attributed to said profiles can be successfully accommodated by response errors (for a general overview, see Birnbaum, 2013).

Both modeling approaches have been applied disjointly in the context of dynamic decision making. In the case of cognitive modeling, the application of DFT has been limited to initial proofs of concept (see Busemeyer et al., 2000; Hotaling, 2020) that do not incorporate an attentional mechanism postulated to play a central role (see Hotaling & Busemeyer, 2012). On the other hand, TE modeling has been applied to streamlined experimental designs in order to characterize specific groups of people (e.g., planners vs. non-planners; Hey & Knoll, 2007). Notably, these modeling efforts have yet to consider more complex characterizations, such as changes in the strategies pursued by individuals throughout the experiment (cf. Birnbaum & Wan, 2020).

In the present work, we will rely on both modeling traditions to characterize data from a new experiment in which participants were presented with a variety of multi-stage decision trees. Crucially, these trees were replicated across blocks, allowing for *choice consistency* to be disentangled from people’s true (perhaps axiom-violating) preference profiles. The resulting choice data from this experiment were then subjected to TE modeling, which offered crude but straightforward insights into the prevalence and temporal stability of preferences consistent with backward induction. Subsequent DFT modeling efforts were then deployed to obtain a more fine-grained picture of underlying processes such as sampling through mental simulation and daydreaming.

## Methods

### Behavioral experiment

#### Ethics

Ethical approval for all experiments was obtained through the institutional review boards of the University of Illinois, Urbana-Champaign.

#### Participants

Fifty participants were recruited from the University of Illinois Urbana-Champaign participant pool with a mean age of 23.82 years; 31 self-reported as female, 17 as male, and two as other. Participants were compensated $8 for completing the study with a chance of winning a $200 Amazon gift card.

#### Procedure

After giving informed consent, participants sat in a computer booth where they received a tutorial familiarizing them with the task. The tutorial walked participants through the types of nodes and tree structures they would see in the experiment. Participants were told that their goal was to maximize the number of points they obtain on each trial, and that each point earned would increase their chances of winning a $200 lottery. There were 40 experimental trials, and the typical session lasted 30 min.

Each trial presented a decision tree on-screen with a circle representing the starting position at DN1 (see Figs. A1-3 and the Online Supplementary Materials (OSM) on the Open Science Framework (OSF)). Choice alternatives (CNs) were represented as boxes that participants were told contained an equal number of red and blue balls. Participants chose to move left or right at DN1 by clicking the mouse on either CN1 or CN2, respectively. An animation then showed a marker moving from DN1 to the chosen CN, where the box opened, and a ball was drawn to determine the CN event (see Fig. A1, OSM). A blue ball would send the participant down the left branch, while a red ball would send them down the right branch. With the marker now located at one of four second-stage DNs (DN2), participants made a second decision by clicking on one of the two alternatives below their DN. For *full trees*, the options were two CNs, while for *half trees*, the options were one CN and one ON. Another animation simulated drawing a ball from the chosen CN, followed by movement of the marker to a final ON.^2^ Participants were shown the number of points they earned on that trial before beginning the next trial. Lastly, *single-stage trees* involved a single choice between two eight-outcome gambles (see Fig. A3, OSM). The marker began at DN1, and participants clicked on one of two CNs below. An animation showed a ball being drawn and the marker moving to the final ON indicated by the ball.

#### Materials and design

The decision trees used in this study were based on Hey and Knoll (2007). We used the following procedure to construct four *tree templates* and many superficially distinct trees. *Template 1* was identical to the tree used by Hey and Knoll (2007) and was an example of a *full tree* (see Fig. A1, OSM) containing five DNs, ten CNs, and 16 ONs. *Templates 2*, *3* and *4* were created by adding 1, 2, or 3, respectively, to the value of each ON in Template 1. We chose this procedure in an effort to create perceptually unique trees that shared a common underlying decisional structure. For additional details, see *Tree Design.pdf* on the OSF.

**Fig. 2:**
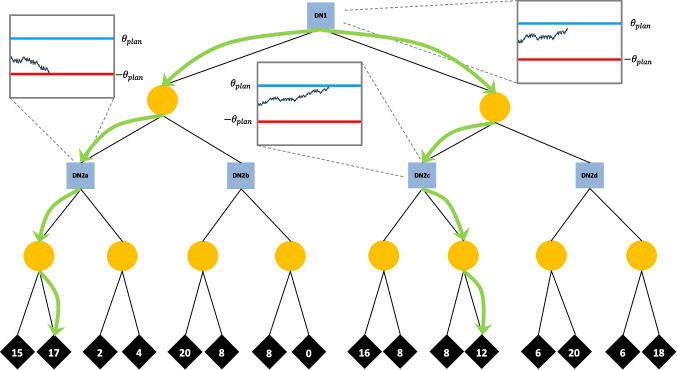
A schematic of Decision Field Theory-Planning (DFT) in a multistage decision tree scenario. The figure captures a snapshot of the DFT 2 version of the model while deliberating at DN1. Green arrows represent the paths of two mental simulations. To simulate the outcome of choosing left versus right at DN1, the model repeatedly traces paths through the tree based on the decision maker’s beliefs. When a simulation reaches a future DN, the model runs an accumulation-to-threshold process to simulate the future decision. Pop-out panels depict the completed simulations at DN2a and DN2c, as well as the ongoing accumulation at DN1. Note that the model uses *θ*_plan_ for DN1 and simulations at DN2, but later uses *θ*_final_ for final choices at DN2 (not shown). Daydreaming – whereby the model randomly samples an outcome – is not depicted in the figure

This structure, which we borrow from Hey and Knoll (2007), is designed to distinguish those who plan ahead from those who do not (see TE modeling section below). To understand why, consider the tree in Fig. 2 in which each DN2 offers a choice between two gambles (i.e., CNs). Crucially, the ON values of one gamble stochastically dominate those of the other such that a participant who plans ahead will easily identify the superior option. For example, moving left at DN2a dominates moving right, and so participants should ignore the possibility of moving right (i.e., prune the right branch) as they consider what to do at DN1. The same pattern holds for the other DN2s. After pruning the appropriate branches, backward induction implies choosing left at DN1 followed by left at DN2. However, trees were designed such that if participants did not plan ahead, the prediction for DN1 reverses. If participants fail to plan, and instead treat DN1 as a choice between two multiple-outcome lotteries – one offering ON1–8 with equal probability and another offering ON9–16 with equal probability (see *forward induction* strategy described below) – moving right stochastically dominates moving left. Thus, non-planners should move right at DN1 and left at DN2.

The procedure for creating four *half tree* templates (see Fig. A2, OSM) began with the full tree templates. At each DN2 we replaced one CN with an ON equal to its expected value. Thus, half trees were the same as full trees, except that DN2 offered a choice between a gamble (CN) and a certain payoff (ON). Each *single-stage tree* template (see Fig. A3, OSM) was also a modified version of a full tree template, in which we essentially removed the second decision stage. The result is a trial presenting a single DN with a choice between CN1 (offering ON1–8 with equal probability) and CN2 (offering ON9–16 with equal probability). Because single-stage trees were created from full trees, one CN stochastically dominates the other and participants are expected to choose the dominant alternative.

Participants completed five blocks of eight trials. Each block involved trials of a single tree type. Each of the four templates was repeated twice per block, with back-to-back repeats disallowed. To obscure our design, and to reduce the role of memory, we scrambled the nodes^3^ on each trial to produce a unique appearance that maintained the tree’s essential dominance properties. Blocks 1–4 alternated between full and half trees, and we used a Latin square design to create four block orders counterbalanced across participants. Block 5 always contained single-stage trees. Participants completed 40 trials, and the typical session lasted 30 min.

### True-and-error modeling

TE modeling of choice data provides us with a first general characterization of the prevalence of axiom violations among individuals. This first characterization will then be used to inform the more fine-grained characterization provided by DFT. One particularly attractive feature of TE models is that, by focusing on choice patterns rather than singular choices, they enable the use of aggregate data without the risk of falling prey to one of many well-documented aggregation fallacies (for a relevant discussion, see Regenwetter & Robinson, 2017). TE models also have the advantage of being deployable over small subsets of trials, such as the two-stage trees, unlike more fine-grained models that call for a richer empirical substrate.

In the present case, the TE modeling conducted focused on the prevalence of choices consistent with backward induction by characterizing the subset of trials most privileged to assess it – two-stage trees. As illustrated in Fig. A1 (OSM), two-stage trees are comprised of two layers of DNs and CNs. These trees were designed such that, at the first DN, an individual engaging in backward induction would have the *opposite preference* to an individual evaluating each side as a multiple-outcome lottery – which we will call *forward induction*. Observing choice patterns for a given two-stage tree was made possible by presenting it once for every test block (with their outcomes reshuffled).

Binary choices made for a given decision tree across four choice-pair presentations can be represented as one out of 16 binary patterns of length 4, with **1** indicating that a binary choice consistent with backward induction was made, and **0** otherwise. These choice patterns are listed in Table 1.

**Table 1:** Binary preference patterns presumed by the true-and-error model

Pattern #	Choice-pair presentation			
	1	2	3	4
1	0	0	0	0
2	1	0	0	0
3	0	1	0	0
4	1	1	0	0
5	0	0	1	0
6	1	0	1	0
7	0	1	1	0
8	1	1	1	0
9	0	0	0	1
10	1	0	0	1
11	0	1	0	1
12	1	1	0	1
13	0	0	1	1
14	1	0	1	1
15	0	1	1	1
16	1	1	1	1

TE models describe the frequency distribution of *choice patterns* through probability parameters. These parameters quantify the probabilities of a randomly sampled participant having a specific *true preference profile* as well as the probability of accurately expressing them through their individual choices. For true preference profiles, let *B* denote the probability that the sampled participant’s preferences are consistent with backward induction. In the absence of errors, the 16th choice pattern 1 1 1 1 is expected (see Table 1). With complementary probability *1*–*B*, the sampled participant’s preferences did not conform to backward induction and instead were consistent with forward induction. In the absence of errors, the first choice pattern of 0 0 0 0 is expected instead (see Table 1).

The assumption of errorless choices is implausible, and therefore relaxed by assuming a so-called “trembling hand” error parameter *e* that can range between 0 and 0.5.^4^ Assuming that errors (but not choices) are independent and identically distributed allows the TE model as developed so far to provide simple closed-formed solutions to the probabilities of each choice pattern. For instance, the probability of choice pattern 1 0 1 1 corresponds to:
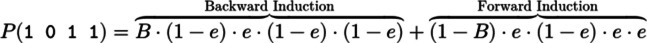


This TE model can be further extended by allowing both preferences and errors to change halfway through the experiment (for a similar approach, see Birnbaum & Bahra, 2012). Let ${s}_{b}$ denote the probability consistency with which backward induction continues in the second half of the experiment (third and fourth presentations of the choice pair), and let ${1-s}_{b}$ denote the complementary probability that there is a *switch* to preferences consistent with forward induction. Conversely, let ${s}_{f}$ denote the probability that consistency with *forward* induction continues to be pursued in the second half of the experiment, and $1-{s}_{b}$ the complementary probability that there is a switch. Moreover, let us assume that the probability of a choice error can change halfway through the experiment by assigning parameters ${e}_{12}$ and ${e}_{34}$ to blocks 1–2 and 3–4, respectively. According to this extended model, the probability of choice pattern 1 0 1 1 corresponds to:
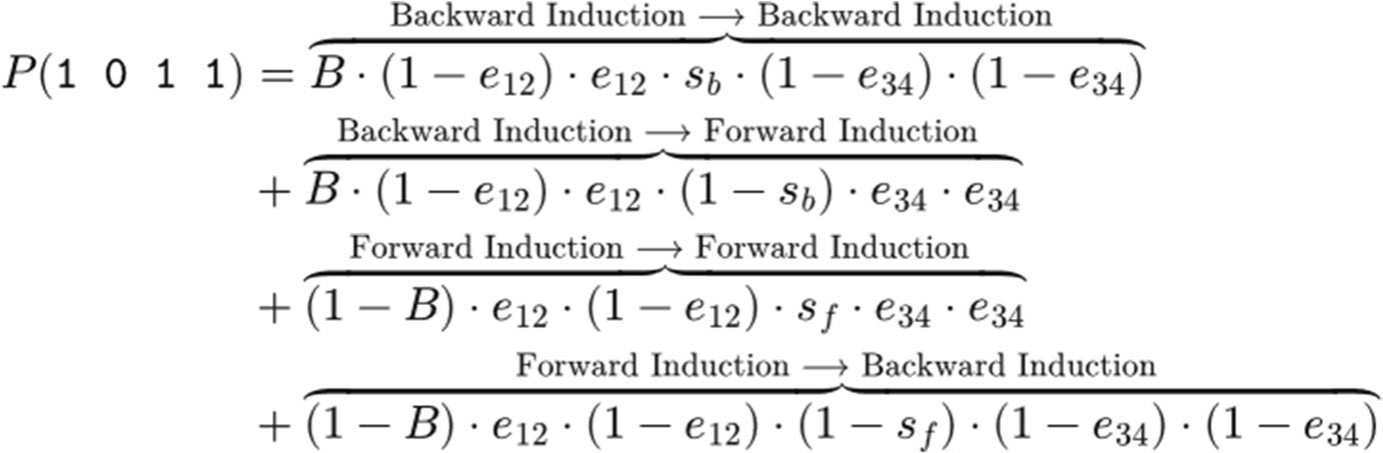


This extended TE model was applied to the choice pattern frequencies obtained across the different two-stage trees. The resulting data provide 15 degrees of freedom on account of the 16 choice patterns, which are far more than the five model parameters postulated: *B*, ${s}_{b}$, ${s}_{f}$, ${e}_{12}$, and ${e}_{34}$.

By virtue of being a member of the class of multinomial processing tree models (Riefer & Batchelder, 1988), the methods for fitting and testing TE models are well established (e.g., Singmann & Kellen, 2013). In the present case, we relied on maximum-likelihood estimation and null-hypothesis testing, although other methods are available (e.g., Bayesian estimation and testing; see Lee, 2018).

### Decision Field Theory-Planning (DFT) modeling

We tested several process models with the aim of discovering the cognitive mechanisms that supported dynamic decision making. By comparing the performance of models with different mechanisms, we can test hypotheses about the information processing capacities and decision making strategies of participants. The present version of DFT (see also Hotaling, 2020; Hotaling & Busemeyer, 2012) extends the model originally developed by Busemeyer and Townsend (1993) to the context of dynamic decision making. According to DFT, the decision maker has a preference state, *P*, signifying the balance of evidence in favor of each option. Preference evolves as people think through complex choice scenarios using a process of noisy mental simulation. Individuals form a mental model of the decision problem – typically assumed to match the decision tree presented to participants – and imagine possible sequences of events that might result from their actions. Each simulation amounts to (virtually) tracing a path through the decision tree from the current DN to a final ON. More formally, at each moment in time, a mental simulation is run for each alternative, *i* and *j*, and the resulting outcomes, ${\nu }_{i}$ and ${\nu }_{j}$, are compared to produce a momentary valence in favor of *i*:

1 $$V= {\nu }_{i}-{\nu }_{j}.$$

The preference state at time *t* is the sum of the previous preference state and the new valence:

2 $$P\left(t\right)= P\left(t-1\right)+V\left(t-1\right).$$

The initial preference is assumed to be unbiased (i.e., *P(0)* = 0).^5^ Deliberation continues – with new simulations producing new valences that are added to the preference state – until the absolute value of preference exceeds a threshold value, *θ*.^6^ If *P*(*t*) > 0, *i* is chosen; if *P*(*t*) < 0, *j* is chosen. In general, the mean input from an alternative is the sum of all possible outcomes, weighted by the likelihood of mentally simulating each. That is, for each alternative, *i*, leading to *k* possible outcomes, ${o}_{ik}$, the mean input to the deliberation process is:

3 $$\overline{{\nu }_{i}}=\sum_{k}S\left({o}_{ik}\right),$$

where *S* is a function specifying the probability of simulating each outcome.

To provide an intuitive overview of DFT and each of its cognitive mechanisms, it is helpful to first consider how it makes simple, two-alternative risky decisions before turning to the more complex dynamic decision making context. Imagine Alternative *A* offers a certain payoff of *X* and Gamble *B* offers *Y* with probability *p*, otherwise *Z*. Assuming no *sampling bias* or *daydreaming* (see below), simulations for *A* will always result in sampling *X*, while simulations for *B* will result in sampling *Y* with probability *p* and *Z* with probability 1-*p*. The mean valence is therefore equal to the difference in mean values across alternatives: $\overline{{\nu }_{A}}-\overline{{\nu }_{B}}=X-EV\left(B\right)=X-(p\bullet Y+(1-p)\bullet Z)$. Thus, the model provides a process account of decision making resulting in preference favoring the higher expected value (EV) alternative. The degree of determinism in the model is controlled by the threshold value, with higher values producing more exhaustive sampling and higher EV maximization, and lower values producing more random choices based on only a few noisy samples.

Returning to the problem faced by Emma in Fig. 1, DFT uses the above equations to calculate predictions for each simple choice at DN2. These equations also hold for DN1, but now each simulation must trace a longer path from DN1 to an ON. At each step in the deliberation process Emma simulates the outcome of moving left – tracing a path through CN1 to one of ON1-8 – and of moving right – tracing a path through CN2 to one of ON9-16. In its simplest form, the probability of simulating a given outcome is the product of three values: ρ_1_ – the transition probability at CN1/2; ρ_2_ – Emma’s choice probability at DN2; and ρ_3_ – the transition probability at CN3-10. For example, let us consider the case of simulating ON1. As Emma imagines moving left at DN1, she first simulates the event at CN1. If she believes her boss has a 60% chance of saying Yes, 60% of simulations will move left and 40% will move right. Assuming Emma simulates moving left at CN1, she will next arrive at DN2a, where she must now imagine her future decision. DFT models this simulated future decision using the same process described above, with Emma’s predicted choice probability determining the likelihood of simulating each alternative. Assuming Emma simulates choosing *company stock* at DN2a, her beliefs about CN3 are the final determinant. She simulates receiving ON1 with probability *q*, otherwise she imagines ON2 with probability 1-*q*, where *q* is Emma’s subjective probability of ON1. Having simulated the outcome of moving left at DN1, Emma repeats the process for moving right, then compares the outcomes (Eq. 1) and integrates the result (Eq. 2). This repeats, with Emma stochastically simulating and comparing outcomes until her preference for one alternative exceeds *θ*. Figure 2 depicts the decision making process of DFT for a decision tree in the experiment (see *DFT Models.pdf* on the OSF for more details). While deliberating over the choice at DN1, the model simulates paths through the tree, and in so doing simulates future choices at DN2. Thus, DFT presents a vision of dynamic decision making that sharply contrasts with that of backward induction. Crucially, planning of future choices is done on-the-fly through repeated, noisy, forward-looking mental simulation, rather than strictly optimal, backward-looking, commitments.

We investigated the contribution of three cognitive mechanisms that aid DFT in explaining dynamic choices. The first mechanism, *threshold shift*, allows the model to process information differently across decision stages by using different decision thresholds for planned and final decisions. Recall that for every timestep in the deliberation process at DN1, Emma must simulate two entire future decisions; one at DN2a/b and another at DN2c/d. Given the large number of simulations required to make a choice, it would be reasonable to use a lower threshold, *θ*_plan_, at DN1 and when simulating future choices at DN2. After making an initial decision at DN1, a higher threshold, *θ*_final_, could be used for the simpler final choice at DN2.

*Sampling bias* is a mechanism drawing on the intuition that, in naturalistic contexts, various environmental, personality, or cognitive factors (e.g., salience, memory strength, emotional significance) will bias mental simulations away from an accurate reflection of objective likelihoods, even when the probabilities are fully described. While many such biases may manifest in real world scenarios, in simple laboratory settings – where outcomes are represented as numerical values – it stands to reason that outcome magnitude is the primary factor biasing attention (see Madan et al., 2014; Vanunu et al., 2020). As such, when DFT simulates an event at CN3-CN10, the higher magnitude outcome is sampled with probability *ϕ*, and the lower magnitude value is sampled with probability 1– *ϕ* (cf. de Gardelle & Summerfield, 2011; Vanunu et al., 2021). Biased sampling is assumed to occur only when planning ahead at DN1 (and simulating future choices for DN2), while final choices at DN2 involve unbiased sampling according to the true CN probabilities (i.e., 50%).

The final mechanism, *daydreaming*, holds that people may sometimes lose focus and let their mind wander to other matters. When this happens, simulations are not limited to the outcomes in the decision tree but are instead drawn from the universe of possible outcomes. We represent this by randomly sampling a value from a uniform distribution spanning the range of ON values in the experiment. With probability *δ* the model samples from $U\left(n,m\right)$, where *n* and *m* are the minimum and maximum values across all trials, respectively. Limiting these daydreams to the range of values found in trees represents the intuition that people do not consider unrealistically extreme outcomes (see Bhatia, 2014, and Kellen et al., 2020, for similar, distracted versions of DFT).

We examined four variants of DFT. Each is built within the shared framework of noisy mental simulation but is designed to represent a unique strategy. *DFT 2* is the most sophisticated model, having separate threshold parameters for each decision stage. *θ*_plan_ is the subjective evidence threshold used for decisions at DN1 and for planned (i.e., simulated) decisions at DN2, whereas *θ*_final_ represents the threshold for final choices at DN2. Estimating parameters separately allows for changes in processing across stages, and we expected *θ*_plan_ < *θ*_final_ for most individuals^7^ – indicating frugality when planning ahead, but increased caution when making final decisions. *DFT 1* is identical to *DFT 2*, but assumes no change in evidence threshold across stages, i.e., *θ*_plan_ = *θ*_final_.

The final two model variants, *DFT 1*_*no-plan*_ and *DFT 2*_*no-plan*_, are *non-planning* versions of the first two. They represent the hypothesis that individuals do not plan future choices, but instead treat future DNs like equiprobable CNs. That is, when deliberating at DN1 and imagining the future event at DN2, these models simply “flip a coin” in lieu of simulating a decision (i.e., ρ_2_ = .50).

As a standard for comparison, we investigated the performance of a flexible version of backward induction, the *baseline* model. However, we exclude this model from the analyses below because it yielded poor qualitative fits to data, and it was never selected as the best model for any individual (see Appendix B in the OSM for more details).

#### DFT modeling procedure

To determine the best model-based characterizations, we perform two complementary analyses. Our primary quantitative analysis relies on cross-validation to determine the model that most successfully predicts individual choices (Busemeyer & Wang, 2000). We also use maximum-likelihood estimation to fit the models to the individuals’ choices. These fits are examined to determine each model’s ability to reproduce key behavioral patterns.

The four-fold cross-validation analysis was conducted as follows: For each individual participant, we randomly allocate trials to four different *training* sets, such that 25% of trials in each condition are part of each (and only one) training set. A corresponding *validation* set is created for each training set, which contains the remaining 75% of trials. For each choice trial, the models’ predicted choice probabilities are estimated by simulating choices from each model 300 times. We assume a binomial error process to connect predicted choice probabilities to observed choices. The best-fitting parameter estimates are then used to predict responses for trials in the validation set. This procedure is followed four times – once for each training set – and the predictive accuracy results are averaged. We use these mean out-of-sample prediction accuracies as our primary goodness-of-fit measure. Comparing this measure across models tests how well each model accounts for the data while also implicitly penalizing undue model complexity (see Busemeyer & Wang, 2000).

## Results

### Behavioral results

Behavioral analyses focused on the proportion of participants’ choices that matched the predictions of the normative backward induction model, i.e., their *maximization rate*. At DN2, the prediction was simply to choose the alternative with the higher expected value. At DN1, the prediction was to choose the CN with the higher expected value, with the values for CN1 and CN2 calculated via backward induction.

Mean maximization rates for final choices at DN2 were high for both full (*M* = 0.96, *SE* = 0.01) and half (*M* = 0.91, *SE* = 0.01) trees, indicating that participants easily identified the high-value alternative when making these simple choices. Choices on single-stage tree trials – which similarly involved immediate outcomes and no planning – were also highly accurate (*M* = 0.91, *SE* = 0.02). In contrast, choices at DN1 showed lower maximization for both full (*M* = 0.44, *SE* = 0.04) and half (*M* = 0.58, *SE* = 0.04) trees. Since a failure to maximize may result from either an error in implementing the backward induction strategy or the use of an alternative strategy, in the following section we use TE modeling to identify the presence of backward- versus forward-working strategies, independent of response errors.

### True-and-error modeling results

We begin with full two-stage trees. In terms of badness of fit, the TE model provided a competent account of the data (${G}_{df=10}^{2}$ = 17.01, *p* =.07).^8^ In terms of parameter estimates, the probability *B* of someone having preferences consistent with backward induction from the start was found to be merely.30. Among these, the probability ${s}_{b}$ of continuing to hold preferences consistent with backward induction in the third and fourth blocks was.81. In the case of forward induction, the analogous probability ${s}_{f}$ was found to be.64. These values suggest a modest increase in the predominance of backward induction, from.30 to.49. Lastly, the choice error probabilities ${e}_{12}$ and ${e}_{34}$ were found to be moderately low, with estimates of and.22 and.15, respectively. To corroborate this characterization, we tested a restricted version of the TE model that assumed that everyone held preferences consistent with backward induction right from the start (i.e., *B* = 1), or that these preferences were maintained in the second half of the experiment (${s}_{f}$ = ${s}_{b}$ = 1). Enormous increases in badness of fit were found for the first two restricted models (smallest ${\Delta G}^{2}$ = 24.64, largest *p* <.001). Altogether, the TE modeling of two-stage lotteries indicates that only a (sizeable) minority of participants have preferences that satisfy backward induction, although its predominance increases as participants continue to encounter full two-stage trees.

We performed the same modeling analysis on the half trees. Once again, the TE model provided a good account of the data (${G}_{df=10}^{2}$ = 7.05, *p* =.72) but this time the estimated probabilities *B* of initiating with preferences consistent with backward induction (.61) and ${s}_{b}$ of preserving them in the second half of the experiment (1.00) were considerably higher. The estimated probability of preserving preferences consistent with forward induction was also found to be larger (0.78). These parameter estimates indicate a small increase in the proportion of individuals consistent with backward induction in the second half of the experiment (.69). Restricting *B* = 1 led to gross misfits (${\Delta G}^{2}$ = 28.75, *p* <.0001) but not ${s}_{f}$ = ${s}_{b}$ = 1 (${\Delta G}^{2}$ = 1.5, *p* =.23). Lastly, the estimated choice error probabilities ${e}_{12}$(.30) and ${e}_{34}$ (.20) were slightly larger than their full-tree counterparts.

The estimated differences in true preferences consistent with backward induction are in line with the higher maximization rates observed in half trees relative to the full trees. To test this difference more rigorously, we fit a TE model in which all preference-related parameters were constrained to be the same across both full and half trees, allowing for choice-error probabilities to differ between the two. The badness of fit of this model was considerable, when compared to an encompassing model that did not impose those parametric constraints (${\Delta G}_{df=2}^{2}$ = 22.99, *p* <.001).

Altogether, the TE modeling results indicate that a considerable portion of participants held preferences consistent with backward induction, albeit with some temporal variation. Once again, note that these preferences are at odds with the notion that participants are simply engaging with the first decision node as a choice between two multiple-outcome lotteries – something else is going on. Our goal then is to go beyond the agnostic characterization offered by TE modeling. We will pursue it by implementing and evaluating a number of DFT models that postulate mental simulation processes that can result in preferences consistent with backward induction.

### DFT modeling results

Our first analysis aimed to select the best and most parsimonious DFT model for each individual using cross-validation. Since the models are meant to represent individuals’ decision strategies across all trials, not just two-stage trees, we focus on the individual-level results. DFT 2 was the best performing model for 19 individuals (38%), followed by DFT 2_no-plan_ with 17 individuals (34%). DFT 1 and DFT 1_no-plan_ performed equally well, and were each the best model for seven individuals (14%). These results indicate that most people (58%) were best characterized by the *threshold shift* mechanism whereby they set different evidence thresholds at DN1 and DN2. A slight majority (52%) planned future choices for DN2, while the others did not.

**Fig. 3:**
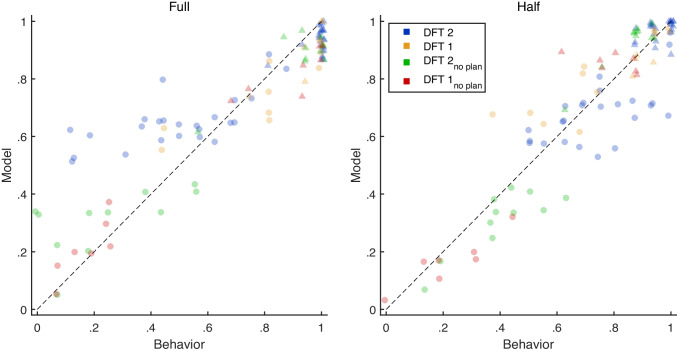
A comparison of observed and predicted individual mean maximization rates at DN1 (triangles) and DN2 (circles) for full (**left panel**) and half (**right panel**) trees. Predictions are shown for each individual’s best model, according to cross-validation

**Fig. 4:**
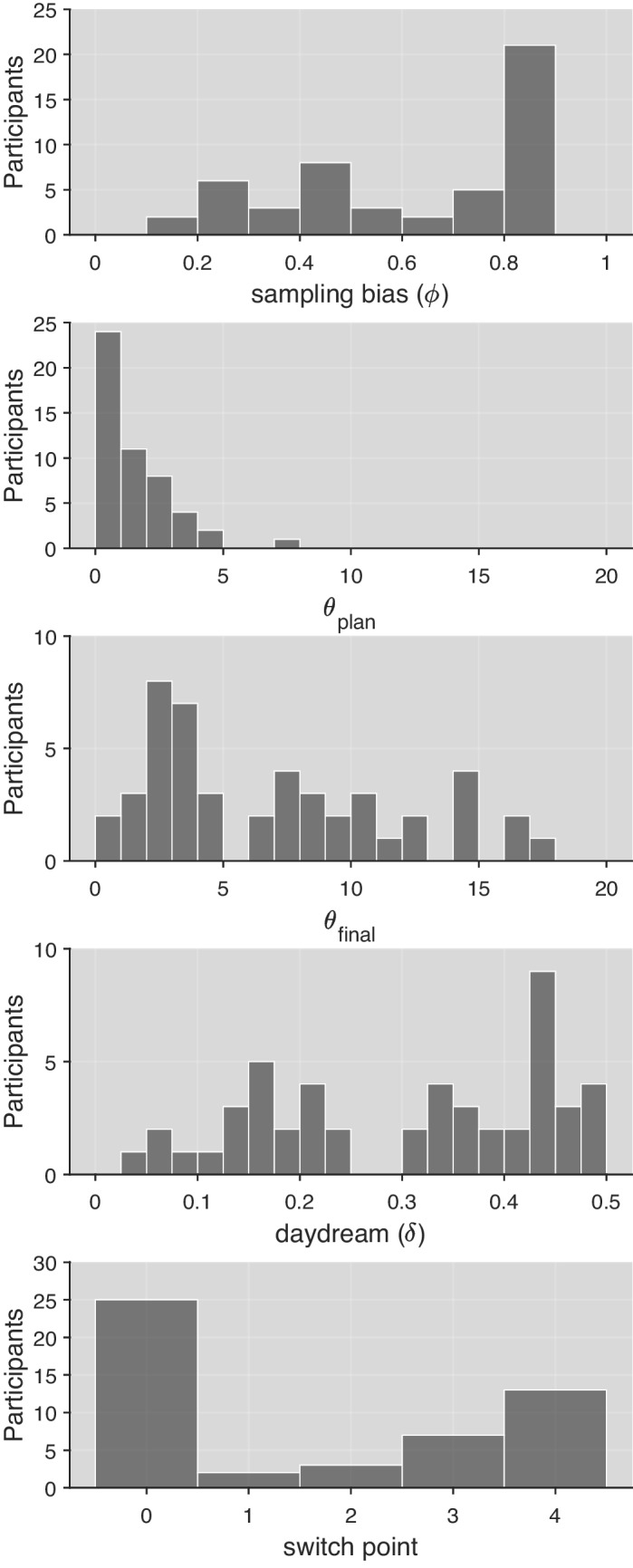
Distributions of individuals’ best-fitting parameters for the full Decision Field Theory-Planning (DFT) model

To better understand the performance of the models, we now turn to a qualitative analysis. We discuss the distributions of parameter estimates for the best-fitting version of DFT below (see Fig. 4). Figure 3 compares observed mean choice proportions to model predictions for full (left panel) and half (right panel) trees. Each individual’s predictions come from the model with the best performance under cross-validation.^9^ Each individual contributes two datapoints to each panel: mean maximization rates are indicated by triangles for DN2 and circles for DN1. Although all of the models do well in predicting behavior at DN2 – where maximization rates are very high – interesting patterns emerge for the more heterogeneous behavior at DN1. Non-planning models (DFT 1_no-plan_ and DFT 2_no-plan_) were best for participants with low maximization (below.5), while planning models (DFT 1 and DFT 2) were preferred for those with higher maximization (above.5). If we consider how the models deliberate at DN1, this pattern is quite sensible: Planning models tend to simulate choosing the superior alternatives in the future and will therefore make choices at DN1 that largely match those predicted by backward induction. In contrast, non-planning models do not simulate maximizing choices – because they are equally likely to imagine choosing either option in the future – and therefore better match individuals who violate the predictions of backward induction. A small number of individuals best fit by DFT 2 show a noteworthy pattern of misfits, with maximization rates higher than predicted for Full trees and lower than predicted for Half trees (blue dots in Fig. 3). The section *Adaptive strategy selection* in the *General discussion* discusses these in the context of *strategy adaptation*, which remains an interesting avenue for future investigations. Readers interested in the patterns of model misfit should consult Fig. A5 in the OSM for the predictions of all models for all individuals.

In light of the TE modeling results suggesting a variation in preferences throughout the experiment, we also considered an extended DFT 2 model that allows for a change in processing – from not planning to planning – during the experiment. A *switch point* parameter was allowed integer values from 0 to 4, and determined the block after which a person began planning. Values of 0 or 4 indicated consistent planning or non-planning, respectively (i.e., no strategy switch). To avoid introducing excessive complexity into the extended model, the model only considered a change from non-planning to planning.^10^

The bottom panel of Fig. 4 shows the distribution of best-fitting *switch point* parameters, revealing a pattern in keeping with our TE findings. Half of participants (25) appeared to plan for the entire experiment – *switch point* of 0 – while13 (26%) never planned – *switch point* of 4. The remaining 12 (24%) individuals did not plan initially, but eventually adopted a planning strategy after Blocks 1, 2, or 3. These results differ somewhat from those obtained with TE modeling, which can be attributed to a number of reasons, such as the fact that the TE model was fit to separate subsets of the choice trials (half and full two-stage trees), whereas DFT considered all choice trials simultaneously. Another important difference is the fact that the TE model only allowed for a change to occur halfway through the experiment, whereas in the DFT model this change could take place in any block.

The other panels of Fig. 4 also provide insights into decision strategies. As expected, the threshold shift mechanism yielded substantially lower values for *θ*_plan_ than *θ*_final_. This indicates that people made quicker, more error-prone decisions when planning and simulating at DN1, but later raised their standards when making careful final decisions at DN2. A range of sampling bias (*ϕ*) values was found, with many individuals showing a strong tendency to simulate the higher magnitude outcome when planning ahead. Daydreaming (*δ*) also showed a range of values, with some people rarely daydreaming and others doing so almost half of the time. Future work evaluating the disposition towards daydreaming is necessary.

## General discussion

Many decision problems in life require people to plan across multiple decision stages. This feature is all but absent in the mainstream study of decision making under risk. The goal of the present work is an attempt to bridge the existing gap and showcase the research potential of dynamic decision making. To illustrate the possibilities available to researchers, we characterized people’s choices using different modeling approaches when investigating their adherence to the normative benchmark that is backward induction.

TE modeling informed us that a small but sizeable portion of the individuals held true preferences consistent with backward induction, with some degree of temporal variation. The preferences are at odds with the notion that participants treat the decision trees as choices between two multiple-outcome lotteries. In other words, these preferences call for an explanation that takes into account the dynamics of the tree and its decision/chance nodes. This is what the DFT modeling provides, by proposing a mental simulation process that can be deployed during planning.

We tested several versions of DFT – each of which used mental simulations to accumulate evidence to a decision threshold – and found clear individual differences. A slight majority (52%) of people planned their future choices before making an initial move, while the others did not and instead treated their future choice as a random chance event. Our model comparison also showed that most individuals (58%) adjusted their information processing across decision stages by using a low evidence threshold while planning ahead at DN1 but setting a higher (i.e., more cautious) threshold for their final decisions at DN2.

The resulting DFT characterization sheds light on some of the processes underlying people’s choices, such as their ability to imagine outcomes (e.g., sampling bias), restrict their attention to the relevant outcomes (e.g., daydreaming), or keep the same strategy throughout the experiment (switch point). Understanding how these characteristics relate with individual (e.g., working-memory capacity, Schapiro et al., 2022) or situational factors (e.g., the psychological distances induced by the description of the decision trees, Trope & Liberman, 2010) strike us as some of many important research avenues that are yet to be pursued.

### Mental simulation

Previous research suggests that mental simulation plays a key role in many behaviors. It follows directly from the idea of *mental sampling* inherent to sequential sampling models (see Hotaling et al., 2024) as well as trace-based models of choice (Gonzalez & Dutt, 2011; Hotaling et al., 2022; Stewart et al., 2006) and memory (Hills et al., 2015). More directly, Kahneman and Tversky's (1982) *Simulation Heuristic* was used to explain how people estimate the likelihood of a counterfactual event based on how easy it is to imagine mentally. Klein and colleagues (Klein, 1993, 1998) proposed the *Recognition-Primed Decision Model* to explain how people make fast decisions in complex, high-stakes scenarios by quickly simulating and selecting the first option deemed satisfactory. Future research should investigate models of dynamic decision making that incorporate non-compensatory or heuristic strategies into broader computational frameworks, such as DFT. Such models would provide new approaches for representing time and effort-saving simplification strategies in complex environments.

Neuroscience also provides evidence for mental simulation. For instance, Johnson and Redish (2007) and Dragoi and Tonegawa (2010) found patterns of neural activity indicating mental simulation in rats navigating mazes in search of food. Pezzulo and colleagues (Chersi & Pezzulo, 2012; Pezzulo et al., 2013) developed a computational model of these tasks in which rats virtually “walk in the hippocampus” by simulating movement along different paths. Using fMRI Suzuki et al. (2012) showed that people use “direct recruitment” of their own mental processes to imagine and predict the choices of others. This suggests another path for future research investigating dynamic decision making strategies in competitive choice scenarios because DFT offers a flexible framework for modeling the cognitive processes involved in planning ahead while also considering the future actions of an opponent.

### Adaptive strategy selection

Research has shown that people modify their decision making strategies in response to task demands (Gigerenzer & Todd, 1999; Glöckner et al., 2014; Payne et al., 1993, Rieskamp & Otto, 2011). The seminal work of Payne et al. (1988) showed that many participants adaptively select strategies in response to changing information structures and time pressure. Although the above experiment was not designed for this purpose, we do find evidence that participants changed their strategies across conditions. Figure 3 shows a small group of individuals for whom DFT 2 overestimates maximization for full trees (blue circles falling above the diagonal) but underestimates maximization for half trees (blue circles falling below the diagonal). This pattern suggests that some individuals changed their approach in a way that is not captured by DFT (or TE models) in its current form – for example, planning for half trees but not for full trees. Examining the role of adaptive strategy selection in dynamic decision making is an important avenue for future research, with the goal of developing process models that capture changes in processing as people encounter and adapt to various types and degrees of complexity.

### Response times

Response times (RTs) are another important area for future research, as these may provide valuable insights into the processes underlying dynamic decision making. DFT provides a convenient framework for exploring RT mechanisms, while also highlighting some of the challenges to this pursuit. For example, in the above analyses DFT is free to estimate threshold values that optimize the models’ fits to choice data. However, given that each simulation at DN1 requires an entire simulated decision at DN2, the current versions of DFT might produce unrealistically long RTs. One solution would be to estimate separate threshold parameters for DN1 and for simulated choices at DN2, in which case the latter could be set extremely low so as to avoid overly long RTs. Alternatively, some scaling factor could be used so that each sample within a simulated decision takes less time than a sample within a real decision. Model comparisons incorporating RT data offer a promising method for testing such hypotheses while also further constraining inferences about other mechanisms of dynamic decision making.

### Modeling toolbox

Both TE and DFT models provided similar pictures showing that a sizeable portion of the participants’ preferences are in line with backward induction. This convergence provides mutual support for these modeling approaches, each with its own set of strengths and weaknesses. TE modeling can be deployed in circumstances where participants only encounter a limited number of decision trees. On the other hand, the characterization that it provides is agnostic when it comes to the cognitive processes involved. To go beyond it, a model like DFT is necessary – but its implementation requires a much richer empirical substrate. Rather than thinking in absolutes, researchers are encouraged to see them as different solutions in their toolbox that can be deployed strategically.

## Supplementary Information

Below is the link to the electronic supplementary material.

Supplementary file1 (DOCX 2.44 MB)

## Data Availability

Data and materials are available on the Open Science Framework (OSF) at https://osf.io/b4ytz/?view_only=dcf66387245c4b959bb458e9acd3c54d.

## Footnotes

1 We use the label DN2 when referring to any and all decisions nodes at the second decision stage, for example, DN2a, DN2b, etc.

2 If a participant chose the certain ON at DN2 of a half tree trial, the marker simply moved to that node.

3 Using the tree in Fig. 1 as an example, several perceptually unique versions could be created. ON1 and ON2 could be swapped to make CN3 appear different. CN3 and CN4 could be swapped to make DN2a appear different. CN1 and CN2 could be swapped to make the entire tree appear different.

4 Allowing for *e* to take on values larger than 0.5 would introduce the strange expectation that taking the opposite of someone's responses better captures their true preferences.

5 Non-zero values of *P*(0) can be used to explain response biases and carry-over effects from past experience or previous decisions.

6 DFT belongs to the broader classes of *random walk* and *sequential sampling models*.

7 Note that we do not constrain *ϑ*_plan_ to be less than *ϑ*_final_, but rather freely estimate them and allow the data to speak for themselves.

8 As a reassurance of the robustness of the reported results, note that the conclusions drawn in this section do not hinge on the use of null-hypothesis significance testing. For instance, they are supported by model selection criteria such as the Akaike Information Criterion (AIC) and the Bayesian Information Criterion (BIC). To illustrate, consider the BIC: for both the full-tree and half-tree data, the penalty term is approximately 5.28 per parameter. Applying this penalization to the reported ${G}^{2}$ and ${\Delta G}^{2}$ statistics reveals that cases yielding non-significant results correspond to instances in which BIC favors the simpler (null) model, whereas cases yielding significant results correspond to instances in which BIC favors the more complex (alternative) model.

9 Qualitative modeling results are based on fitting each model to all responses at the individual level. The procedure for estimating optimal parameter values was otherwise identical to that used for the cross-validation analysis.

10 We also tested a version of the model that switched from planning to not planning, but this model produced inferior fits for the majority of individuals.

## Code availability

Modeling code is available on the OSF at https://osf.io/b4ytz/?view_only=dcf66387245c4b959bb458e9acd3c54d.

## Declarations

### Open Practices Statement

Data and materials are available on the OSF at https://osf.io/b4ytz/?view_only=dcf66387245c4b959bb458e9acd3c54d. The experiment was not preregistered.

## References

[CR1] Barkan, R., & Busemeyer, J. R. (1999). Changing plans: Dynamic inconsistency and the effect of experience on the reference point. *Psychonomic Bulletin & Review,**6*(4), 547–554. 10682196 10.3758/bf03212962

[CR2] Barkan, R., & Busemeyer, J. R. (2003). Modeling dynamic inconsistency with a changing reference point. *Journal of Behavioral Decision Making,**16*(4), 235–255.

[CR3] Bertsekas, D. P. (1996). *Dynamic programming and optimal control*. Athena Scientific.

[CR4] Bhatia, S. (2014). Sequential sampling and paradoxes of risky choice. *Psychonomic Bulletin & Review,**21*(5), 1095–1111. 24898202 10.3758/s13423-014-0650-1

[CR5] Birnbaum, M. H. (2008). New paradoxes of risky decision making. *Psychological Review,**115*(2), 463–501. 18426300 10.1037/0033-295X.115.2.463

[CR6] Birnbaum, M. H. (2013). True-and-error models violate independence and yet they are testable. *Judgment and Decision Making,**8*(6), 717–737.

[CR7] Birnbaum, M. H. (2019). Bayesian and frequentist analysis of true and error models. *Judgment and Decision Making,**14*, 608–616.

[CR8] Birnbaum, M. H., & Bahra, J. P. (2012). Separating response variability from structural inconsistency to test models of risky decision making. *Judgment and Decision Making,**7*, 402–426.

[CR9] Birnbaum, M. H., & Schmidt, U. (2008). An experimental investigation of violations of transitivity in choice under uncertainty. *Journal of Risk and Uncertainty,**37*, 77–91.

[CR10] Birnbaum, M. H., & Wan, L. (2020). MARTER: Markov true and error model of drifting parameters. *Judgment and Decision Making,**15*(1), 47–73.

[CR11] Bone, J., Hey, J. D., & Suckling, J. (2009). Do people plan? *Experimental Economics,**12*, 12–25.

[CR12] Busemeyer, J. R., Pothos, E. M., Franco, R., & Trueblood, J. S. (2011). A quantum theoretical explanation for probability judgment errors. *Psychological Review,**118*(2), 193. 21480739 10.1037/a0022542

[CR13] Busemeyer, J. R., & Townsend, J. T. (1993). Decision field theory: A dynamic-cognitive approach to decision making in an uncertain environment. *Psychological Review,**100*(3), 432. 8356185 10.1037/0033-295x.100.3.432

[CR14] Busemeyer, J. R., & Wang, Y. M. (2000). Model comparisons and model selections based on generalization criterion methodology. *Journal of Mathematical Psychology,**44*(1), 171–189. 10733863 10.1006/jmps.1999.1282

[CR15] Busemeyer, J. R., Weg, E., Barkan, R., Li, X., & Ma, Z. (2000). Dynamic and consequential consistency of choices between paths of decision trees. *Journal of Experimental Psychology: General,**129*, 530–545. 11142867 10.1037//0096-3445.129.4.530

[CR16] Chersi, F., & Pezzulo, G. (2012). Using hippocampal-striatal loops for spatial navigation and goal-directed decision-making. *Cognitive Processing,**13*(1), 125–129. 10.1007/s10339-012-0475-7 22806662

[CR17] DeGroot, M. H. (1970). *Optimal statistical decisions*. McGraw-Hill.

[CR18] Dragoi, G., & Tonegawa, S. (2010). Preplay of future place cell sequences by hippocampal cellular assemblies. *Nature,**469*(7330), 397–401. 21179088 10.1038/nature09633 PMC3104398

[CR19] de Garlle, V., & Summerfield, C. (2011). Robust averaging during perceptual judgment. *Proceedings of the National Academy of Sciences of the United States of America,**108*(32), 13341–13346. 21788517 10.1073/pnas.1104517108 PMC3156162

[CR20] Gigerenzer, G., Todd, P. M., The ABC Research Group. (1999). *Simple heuristics that make us smart*. Oxford University Press.

[CR21] Glöckner, A., Hilbig, B. E., & Jekel, M. (2014). What is adaptive about adaptive decision making? A parallel constraint satisfaction account. *Cognition,**133*(3), 641–666. 25243773 10.1016/j.cognition.2014.08.017

[CR22] Gonzalez, C., & Dutt, V. (2011). Instance-based learning: Integrating sampling and repeated decisions from experience. *Psychological Review,**118*(4), 523–551. 21806307 10.1037/a0024558

[CR23] Hey, J. D., & Knoll, J. A. (2007). How far ahead do people plan? *Economics Letters,**96*(1), 8–13.

[CR24] Hey, J. D., & Lotito, G. (2009). Naive, resolute or sophisticated? A study of dynamic decision making. *Journal of Risk and Uncertainty,**38*, 1–25.

[CR25] Hilbig, B. E., & Moshagen, M. (2014). Generalized outcome-based strategy classification: Comparing deterministic and probabilistic choice models. *Psychonomic Bulletin & Review,**21*, 1431–1443. 24865279 10.3758/s13423-014-0643-0

[CR26] Hills, T. T., Todd, P. M., & Jones, M. N. (2015). Foraging in semantic fields: How we search through memory. *Topics in Cognitive Science,**7*(3), 513–534. 26097107 10.1111/tops.12151

[CR27] Hotaling, J. M. (2020). Decision field theory-planning: A cognitive model of planning on the fly in multistage decision making. *Decision,**7*(1), 20–42.

[CR28] Hotaling, J. M., & Busemeyer, J. R. (2012). DFT-d: A cognitive-dynamical model of dynamic decision making. *Synthese,**189*, 67–80.

[CR29] Hotaling, J. M., Busemeyer, J. R., & Rieskamp, J. (2024). Psychological research and theories of preferential choice. In S. Hess & A. Daly (Eds.), *Handbook of Choice Modeling* (2nd ed.). Cheltenham: Edward Elgar.

[CR30] Hotaling, J. M., Donkin, C., Jarvstad, A., & Newell, B. R. (2022). MEM-EX: An exemplar memory model of decisions from experience. *Cognitive Psychology,**138*, 101517. 36116240 10.1016/j.cogpsych.2022.101517

[CR31] Hotaling, J. M., Fakhari, P., & Busemeyer, J. R. (2015). Dynamic decision making. In J. D. Wright (Ed.), *International Encyclopedia of the Social and Behavioral Sciences* (2nd ed., pp. 709–714). Elsevier.

[CR32] Hotaling, J. M., & Kellen, D. (2022). Dynamic decision making: Empirical and theoretical directions. In K. D. Federmeier (Ed.), *Psychology of Learning and Motivation: Advances in Research and Theory* (Vol. Vol. 76, pp. 207–238). Academic Press.

[CR33] Johnson, A., & Redish, A. D. (2007). Neural ensembles in CA3 transiently encode paths forward of the animal at a decision point. *The Journal of Neuroscience,**27*(45), 12176–12189. 17989284 10.1523/JNEUROSCI.3761-07.2007 PMC6673267

[CR34] Johnson, J. G., & Busemeyer, J. R. (2001). Multiple-stage decision-making: The effect of planning horizon length on dynamic consistency. *Theory and Decision,**51*, 217–246.

[CR35] Kahneman, D., & Tversky, A. (1979). Prospect theory: An analysis of decision under risk. *Econometrica,**47*(2), 363–391.

[CR36] Kahneman, D., & Tversky, A. (1982). The simulation heuristic. In D. Kahneman, P. Slovic, & A. Tversky (Eds.), *Judgment under Uncertainty: Heuristics and Biases* (pp. 201–208). Cambridge University Press.

[CR37] Karabatsos, G. (2005). The exchangeable multinomial model as an approach to testing deterministic axioms of choice and measurement. *Journal of Mathematical Psychology,**49*(1), 51–69.

[CR38] Kellen, D. (2019). A model hierarchy for psychological science. *Computational Brain & Behavior,**2*, 160–165.

[CR39] Kellen, D., & Klauer, K. C. (2020). Theories of the wason selection task: A critical assessment of boundaries and benchmarks. *Computational Brain & Behavior,**3*, 341–353.

[CR40] Kellen, D., Steiner, M. D., Davis-Stober, C. P., & Pappas, N. R. (2020). Modeling choice paradoxes under risk: From prospect theories to sampling-based accounts. *Cognitive Psychology,**118*, 101258. 32058123 10.1016/j.cogpsych.2019.101258

[CR41] Klein, G. A. (1993). A recognition-primed decision (RPD) model of rapid decision making. *Decision Making in Action: Models and Methods,**5*(4), 138–147.

[CR42] Klein, G. A. (1998). *Sources of power: How people make decisions*. The MIT Press.

[CR43] Lee, M. D. (2018). Bayesian methods for analyzing true-and-error models. *Judgment and Decision Making,**13*, 622–635.

[CR44] Loomes, G., & Sugden, R. (1982). Regret theory: An alternative theory of rational choice under uncertainty. *The Economic Journal,**92*(368), 805–824.

[CR45] Madan, C. R., Ludvig, E. A., & Spetch, M. L. (2014). Remembering the best and worst of times: Memories for extreme outcomes bias risky decisions. *Psychonomic Bulletin & Review,**21*, 629–636. 24189991 10.3758/s13423-013-0542-9

[CR46] McCausland, W. J., & Marley, A. A. J. (2014). Bayesian inference and model comparison for random choice structures. *Journal of Mathematical Psychology,**62*, 33–46.

[CR47] Payne, J. W., Bettman, J. R., & Johnson, E. J. (1988). Adaptive strategy selection in decision making. *Journal of Experimental Psychology: Learning, Memory, and Cognition,**14*, 534–552.

[CR48] Payne, J. W., Bettman, J. R., & Johnson, E. J. (1993). The adaptive decision maker. The adaptive decision maker (vol. xiii). New York, NY: Cambridge University Press.

[CR49] Pezzulo, G., Rigoli, F., & Chersi, F. (2013). The Mixed Instrumental Controller: Using Value of Information to Combine Habitual Choice and Mental Simulation. *Frontiers in Psychology, 4*. 10.3389/fpsyg.2013.00092 PMC3586710 23459512

[CR50] Regenwetter, M., & Robinson, M. M. (2017). The construct–behavior gap in behavioral decision research: A challenge beyond replicability. *Psychological Review,**124*(5), 533–550. 28504522 10.1037/rev0000067

[CR51] Riefer, D. M., & Batchelder, W. H. (1988). Multinomial modeling and the measurement of cognitive processes. *Psychological Review,**95*(3), 318–339.

[CR52] Rieskamp, J., & Otto, P. E. (2011). SSL: A theory of how people learn to select strategies. *Heuristics: The Foundations of Adaptive Behavior,**135*(2), 207–236. 10.1037/0096-3445.135.2.207 16719651

[CR53] Schapiro, K., Josić, K., Kilpatrick, Z. P., & Gold, J. I. (2022). Strategy-dependent effects of working-memory limitations on human perceptual decision-making. *eLife,**11*, Article e73610. 35289747 10.7554/eLife.73610 PMC9005192

[CR54] Singmann, H., & Kellen, D. (2013). MPtinR: Analysis of multinomial processing tree models in R. *Behavior Research Methods,**45*, 560–575. 23344733 10.3758/s13428-012-0259-0

[CR55] Stewart, N., Chater, N., & Brown, G. D. A. (2006). Decision by sampling. *Cognitive Psychology,**53*, 1–26. 16438947 10.1016/j.cogpsych.2005.10.003

[CR56] Sugden, R. (2005). Experiments as exhibits and experiments as tests. *Journal of Economic Methodology,**12*(2), 291–302.

[CR57] Suzuki, S., Harasawa, N., Ueno, K., Gardner, J. L., Ichinohe, N., Haruno, M., Gardner, J., Cheng, K., & Nakahara, H. (2012). Learning to simulate others’ decisions. *Neuron,**74*(6), 1125–1137. 22726841 10.1016/j.neuron.2012.04.030

[CR58] Trope, Y., & Liberman, N. (2010). Construal-level theory of psychological distance. *Psychological Review,**117*(2), 440. 20438233 10.1037/a0018963 PMC3152826

[CR59] Vanunu, Y., Hotaling, J. M., & Newell, B. R. (2020). Elucidating the differential impact of extreme-outcomes in perceptual and preferential choice. *Cognitive Psychology*. 10.1016/j.cogpsych.2020.101274 32062088 10.1016/j.cogpsych.2020.101274

[CR60] Vanunu, Y., Hotaling, J. M., Le Pelley, M. E., & Newell, B. R. (2021). How top-down and bottom-up attention modulate risky choice. *Proceedings of the National Academy of Sciences of the United States of America*. 10.1073/pnas.2025646118 34561303 10.1073/pnas.2025646118 PMC8488801

[CR61] Von Winterfeldt, D., & Edwards, W. (1986). *Decision analysis and behavioral research* (Vol. 604). Cambridge University Press.

