# Supplementary material for: Toward the cognitive modeling of dynamic decision making
Source: Psychon Bull Rev. 2026 Apr 2;33(4):127. doi: 10.3758/s13423-025-02814-2 (PMC13046585; doi:10.3758/s13423-025-02814-2)
Supplement: Supplementary file 1 — Supplementary file1 (DOCX 2.44 MB) [file 13423_2025_2814_MOESM1_ESM.docx]

**Appendix A**

**1. Decision Tree Trial Types**

*
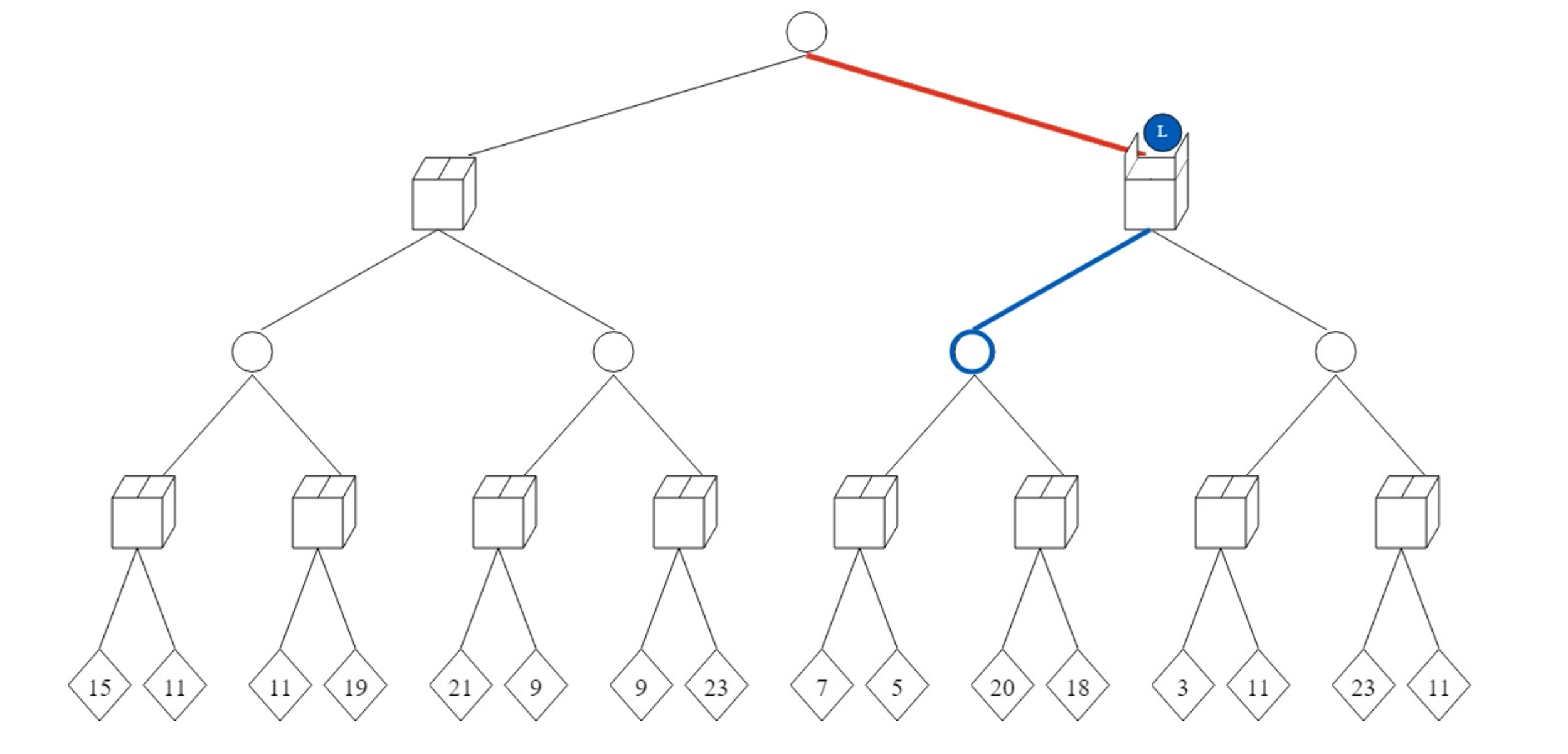
*

*Figure A1*. An example of a full tree in the experiment. The participant chose to move right at DN1. A blue ball was drawn at CN1, and the participant’s current position is DN2c.


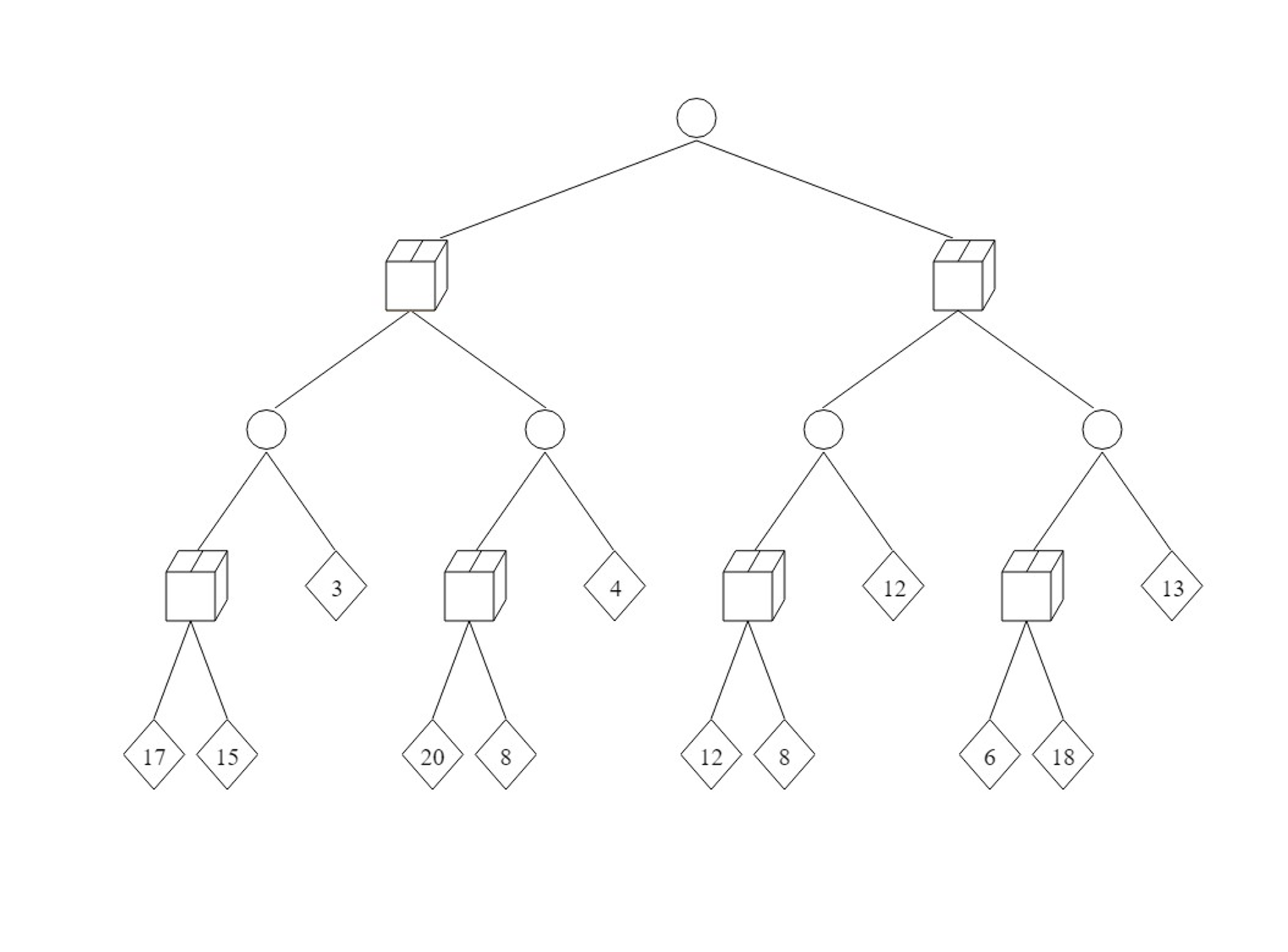


*Figure A2*. An example of a half tree in the experiment.


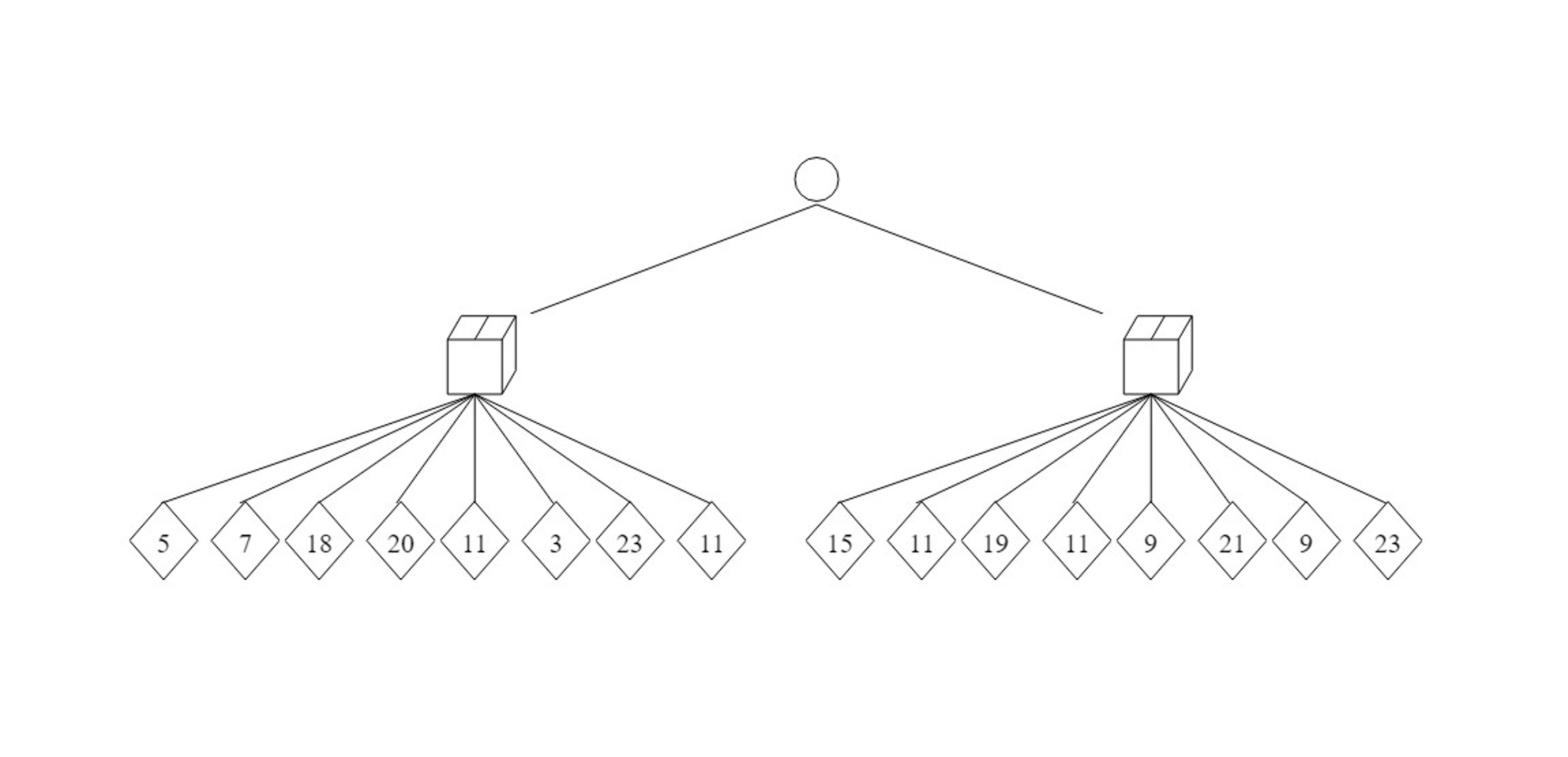


*Figure A3*. An example of a single-stage tree in the experiment.

**Appendix B**

**1. Baseline Model**

The *baseline model* tests whether a flexible version of backward induction can account for behavior when applied to individual-level data using the same procedure as the DFT process models (see Section 2.3.1.). It also provides a reasonable standard for gauging the performance of DFT models. The baseline model is a generalization of backward induction that assumes individuals choose probabilistically according to the logistic choice rule:

|  | $\frac{1}{1+e^{-sd}}$ |  |
| --- | --- | --- |

where *d* is the difference in expected utility across alternatives and *s* is a sensitivity parameter. ON values, *x*, are transformed into subjective utilities using a standard power function, like in prospect theory (Kahneman & Tversky, 1979), where u(*x*) = *x^α^*.^[[1]](#footnote-1)^ *α* is a parameter representing risk attitude, with values below 1 producing risk-aversion and values above 1 producing risk-seeking.

For choices at DN2, the baseline model simply multiplies utilities by their corresponding CN probabilities to compute the expected utility for each alternative. For DN1, the model uses a probabilistic version of backward induction to compute the expected utilities of CN1 and CN2. Unlike the strictly deterministic version of backward induction, the baseline model incorporates uncertainty about future choices. Rather than assuming that all future actions will maximize expected utility, it weights CNs according to their likelihoods of being chosen (i.e. according to the previously computed choice probabilities for DN2). Thus, when *s* is large the static model reduces to the optimal backward induction model with nonlinear risk preference. The model uses two free parameters: *α* and *s*.

Figure B1 compares observed mean choice proportions to the baseline model’s predictions for full (left panel) and half (right panel) trees. Each individual contributes two datapoints to each panel: mean maximization rates are indicated by triangles for DN2 and circles for DN1. Overall, results indicate a poor fit to behavior. Notably, the model cannot produce maximization rates below 0.5, and therefore fails to explain the behavior of many individuals at DN2. At the same time, the model substantially underestimates performance at DN1, where observed maximization rates were very high.


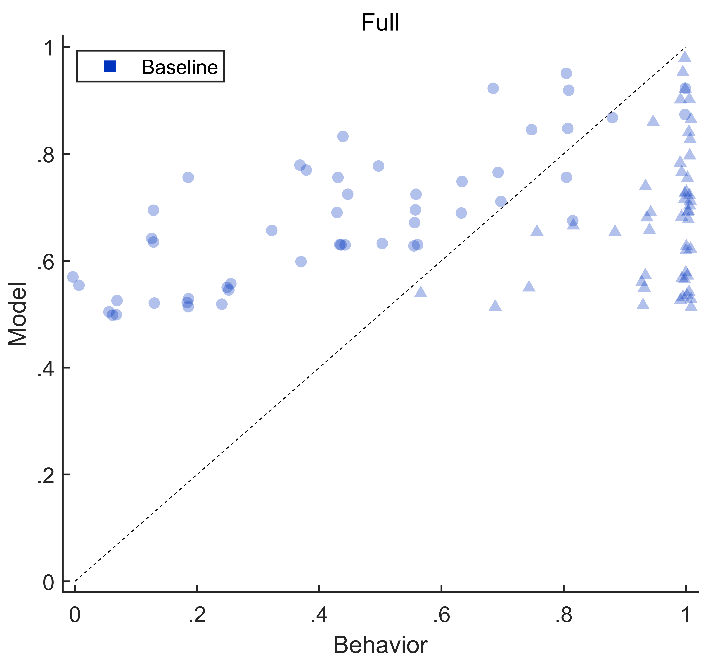

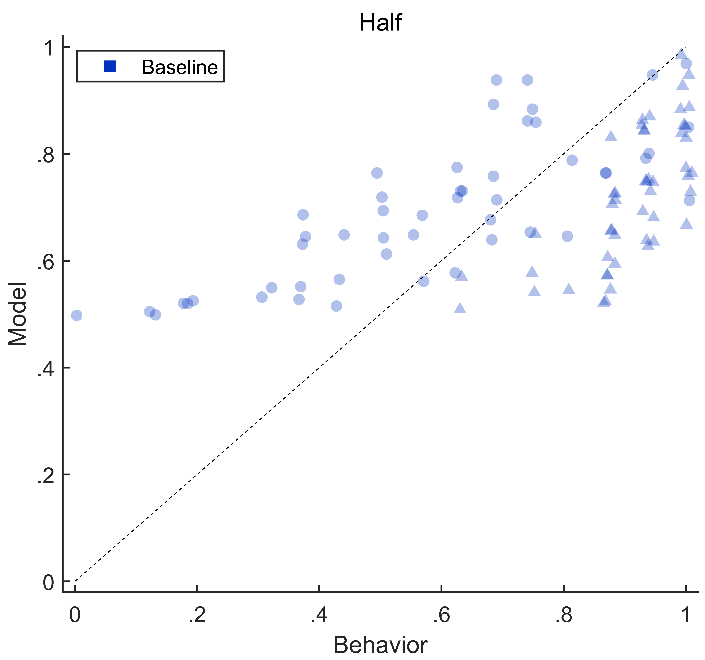


*Figure B1*. A comparison of observed and predicted individual mean maximization rates at DN1 (triangles) and DN2 (circles) for full (left panel) and half (right panel) trees.

**Appendix C**

**1. Process Modeling Results**

Figure C1 visualizes the relative performance of models under cross-validation. To quantify the evidence for each model we computed *model weights* by converting individual mean (across cross-validation folds) log-likelihoods to probabilities such that the weight for Model *i* in the set of *j* models was calculated as $e^{{LL}_{i}}/\sum_{j} e^{{LL}_{j}}$.


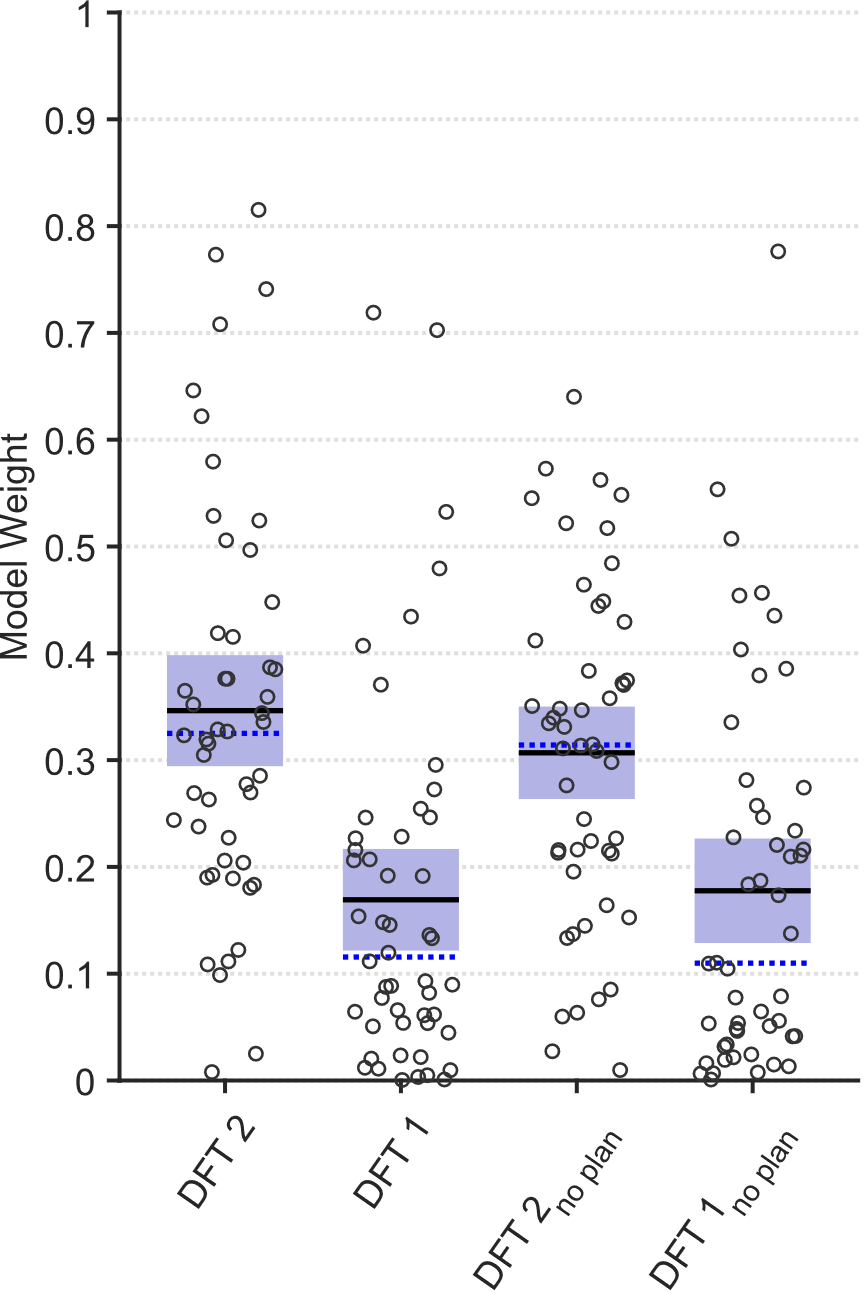


Figure C1. Cross-validation model weights. Each dot represents an individual. Group mean and median values are indicated by the solid and dotted lines, respectively. Shaded bands indicate 95% confidence intervals.

Figure C2 compares observed mean choice proportions to model predictions for full (left panel) and half (right panel) trees. Each individual contributes eight datapoints to each panel; two for each model. Triangles indicate mean maximization rates at DN2, while circles a DN1 maximization rates. It is immediately apparent that each model struggles to account for the behavior of some individuals. Notably the planning models (DFT 1 and DFT 2) do well for individuals who mostly maximize at DN1, but cannot produce maximization rates below .5. A mirror image can be seen in the non-planning models (DFT 1_no-plan_ and DFT 2_no-plan_), which are accurate for low-performing individuals, but struggle to produce maximization rates above .5. These results support and elucidate our cross-validation results by showing that the presence of particular cognitive mechanisms was key for determining which model was selected.


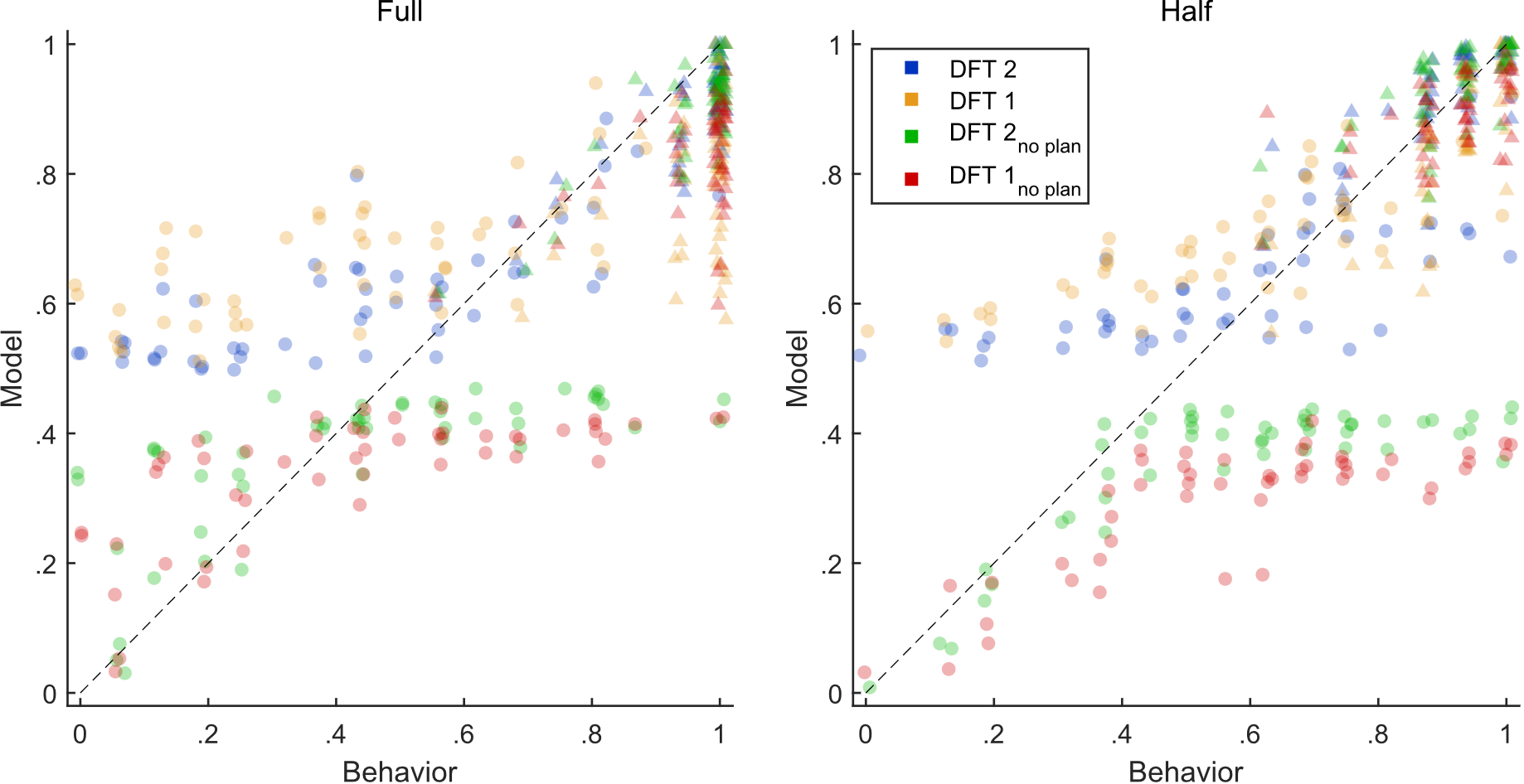


*Figure C2.* A comparison of observed and predicted individual mean maximization rates at DN1 (triangles) and DN2 (circles) for full (left panel) and half (right panel) trees.

**Appendix D**

**1. Model Recovery Analysis**

**1. Method**

We used DFT 2 to investigate model recovery. We began with the best-fitting parameter values for each of the 50 participants and used these to simulate behavior in our experimental design. We repeated this process 10 times for each participant – i.e. each set of parameters was run through the experiment 10 times – to produce a total of 500 simulated agents. We then treated each agent as a participant, and fit DFT 2 to its simulated choices. The analysis below compares the original parameter values from the 50 participants to the recovered values from the 500 simulated agents.

**2. Results & Discussion**

Our primary goal is to draw conclusions about the cognitive mechanisms of dynamic decision making. We therefore focus our recovery analysis on DFT 2’s central component, *threshold shift*. In our initial fits, we found that individuals made quick decisions based on a limited number of mental simulations when planning ahead at DN1, but they were more careful when making final decisions at DN2. Our main recovery analysis shows that we successfully recover this pattern. In our original fitting, *ϑ_plan_* < *ϑ_final_* for forty-nine individuals (98%), and we recovered the same proportion across our 500 simulated agents^[[2]](#footnote-2)^. The magnitude of threshold shift was also similar for the original (*Mdn* = 5.75, *SD* = 3.00) and recovered parameters sets (*Mdn* = 7.00, *SD* = 3.31). Figure D1 shows the relationship between original and recovered threshold shifts at the individual-level. The correlation was moderately high and very significant (*r* = .66, *p* < .001). We therefore succeed in recovering the key mechanism for DFT 2’s explanation of dynamic decision making behavior.


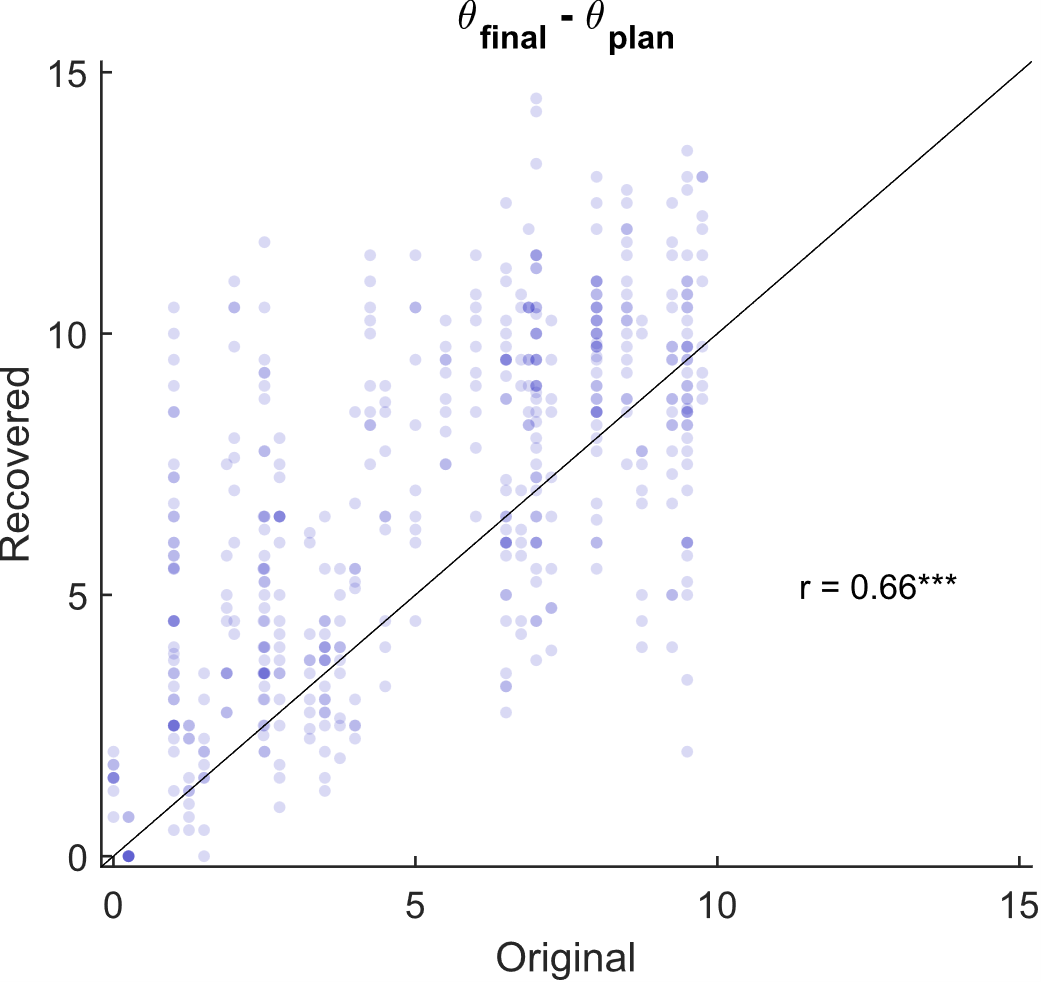


Figure D1. Comparison of threshold parameter differences in original and recovered versions of DFT 2. ****p* < .001

We also examined the recovery of DFT 2’s other parameters despite these being less central to our conclusions. These results are less promising and suggest shortcomings in the model’s ability to produce noisy behavior. Table D1 summarizes the results. Correlations between original and recovered parameters were low for *ϕ* (*r* = .06, *n.s.*) and *δ* (*r* = .13, *p* < .01). We see two potential interpretations of this result. First, it may be that the model’s sampling bias and daydreaming mechanisms trade-off such that different combinations produce similar behavior. Second, the effects of decision thresholds may easily swamp the impacts of other mechanisms. This is easy to imagine for high thresholds, which will tend to yield high maximization because even noisy simulations will typically produce mean valences in favor of the higher EV option. Similarly, very low thresholds will tend to yield random behavior, even when daydreaming and sampling bias are low. Also, our difficulty in recovering *ϑ_plan_* values is of little concern when viewed in the wider context of the threshold shift mechanism where it is the values of thresholds relative to one another that is crucial. In sum, we do not believe that these model recovery results appreciably impact the key insights provided by our process modeling.

| Table D1. *Median original and recovered parameters for DFT 2. Standard deviations are shows in parentheses. r = correlation coefficient. **p* < .01. ****p* < .001. | | | | |
| --- | --- | --- | --- | --- |
|  |  |  |  |  |
| *Model Recovery for DFT 2* | | | |  |
|  |  |  |  |  |
| Parameter | Original | Recovered | *r* |  |
| *ϕ* | . 800 (.197) | .800 (.073) | .061 |  |
| *ϑ_plan_* | .500 (.398) | .500 (.138) | -0.032 |  |
| *ϑ_final_* | 6.625 (2.970) | 7.50 (3.313) | .705*** |  |
| *δ* | 0.388 (.161) | 0.450 (.047) | .134** |  |

1. Note that, because all ON values were positive, there were no *losses* in the experiment. [↑](#footnote-ref-1)
2. We recovered equal thresholds for 1.4% of agents, and *ϑ_plan_* > *ϑ_final_* for .6% of agents, compared to 2% and 0%, respectively, in the original fits. [↑](#footnote-ref-2)
